# Supplementary material for: Structural and Biochemical Analysis of OrfG: The VirB8-like Component of the Conjugative Type IV Secretion System of ICESt3 From Streptococcus thermophilus
Source: Front Mol Biosci. 2021 Mar 18;8:642606. doi: 10.3389/fmolb.2021.642606 (PMC8012802; doi:10.3389/fmolb.2021.642606)
Supplement: Supplementary file 1 [file datasheet1.pdf]

Table S1. Strains, plasmids and oligonucleotides used in this project

Strains		
**Name**	**Genotype**	**Source/reference**
Streptococcus thermophilus	LMG18311 ICE*St3*	Laboratory collection
DH5α	*F-, Δ(argF-lacZ)U169, phoA, supE44, Δ(lacZ)M15, relA, endA, thi, hsdR*	New England Biolabs
BL21 (DE3)	*F- ompT gal dcm lon hsdSB(rB- mB-) λ(DE3)*	Invitrogen
Plasmids		
**Name**	**Description**	**Source/reference**
pET28a-TRX	6His-tag-TEV protease sequences inserted into pET28a+	Laboratory collection
pET28-TRX-OrfG$_{64-331}$	*orfG$_{64-331}$* cloned in frame of 6His-TEV in pET28-TRX	This project
pET28-TRX-OrfG$_{64-204}$	*orfG$_{64-204}$* cloned in frame of 6His-TEV in pET28-TRX	This project
pET28-TRX-OrfG$_{64-215}$	*orfG$_{64-215}$* cloned in frame of 6His-TEV in pET28-TRX	This project
pET28-TRX-OrfG$_{223-331}$	*orfG$_{223-331}$* cloned in frame of 6His-TEV in pET28-TRX	This project

**Oligonucleotides**	
**For cloning in pET28-TRX***	
OrfG-BamHI-F	CCCCGGATCC*GAGAACCTGTACTTCCAATCA*TCAAATAAAGTGGGAACACTAC
OrfG-HindIII-R	CCCCAAGCTTCTAATCTTTATCGTTTGCGTAG
OrfG$_C$-BamHI-F	CCCCGGATCC*GAGAACCTGTACTTCCAATCA*GCCAACGACCACGTCTCAG
*Restrictions sites are underlined and TEV cleavage encoding sequence are highlighted in italics	
**Cloning by site directed mutagenesis (Stop codon insertion by PCR)***	
OrfG$_{64-204}$-Stop-F	ACTTCCTTGGTTCTCAGCGTAAGAGTCTTCACAAGCTGGATAC
OrfG$_{64-204}$-Stop-R	GTATCCAGCTTGTGAAGACTCTTACGCTGAGAACCAAGGAAGT
OrfG$_{64-215}$-Stop-F	AGCTGGATACTTTAGCGAATAGGATCAACTCCAACTCACAG
OrfG$_{64-215}$-Stop-R	CTGTGAGTTGGAGTTGATCCTATTCGCTAAAGTATCCAGCT
* Mutagenized codons are underlined	

Tables S2. Pairwise alignment lengths, identities and similarities between the 13 Gram-negative VirB8 and 4 Gram-positive VirB8-like proteins considered in this comparison **(Table S3a)**. The sequences are derived from the structures, i.e. there might be missing residues (see protein description below) corresponding to unobserved regions in the electron density. Values are calculated from the alignment of the sequences corresponding to the overall superposition of the 17 structures, as performed by mTM-align (Dong *et al.*, 2018a). The TM-scores (Dong *et al.*, 2018b) and the pairwise rmsd (Å) calculated from the same overall superposition are also given **(Tables S3b and S3c)**. Clusters that appear in the structural tree are coloured as in Fig.5 and numbered for their convenient description in the main text.

For each following PDB code, the source organism, the Uniprot code, the sequence that was used for the experiment (X-Ray or NMR), where appropriate the unobserved residues (which are therefore missing in the sequence) and the resulting coverage of the whole sequence are given hereafter.

2cc3: VirB8 from *Agrobacterium tumefaciens*; Uniprot: P17798; Crystallized sequence: 92-237; C-ter (232-237) unobserved in the electron density; Coverage 59%.

3ub1: TcpC from *Clostridium perfringens*; Uniprot: Q1PLH8; Crystallized sequence: 99-309, central domain as defined by authors: 104-231; loop β2-β3 (190-192) unobserved in the electron density (coverage 35%).

3wz3: TraM from Plasmid R64; Uniprot Q9R2H2; Crystallized sequence: 91-230; C-ter (227-230) unobserved in the electron density (coverage 59%).

3wz4: IcmL/DotI from *Legionella pneumophila*; Uniprot: O54626; Crystallized sequence: 73-212; C-ter (209-212) unobserved in the electron density (coverage 64%).

4akz: VirB8 from *Brucella suis*; Uniprot: Q7CEG3; Crystallized sequence: 97-234 (coverage 57%).

4ec6: TraM from *Enterococcus faecalis*; Uniprot: Q8L1C7; Crystallized sequence: 190-322; N-ter (190-213) unobserved in the electron density (coverage 34%).

4jf8: TrwG from *Bartonella birtlesii*; Uniprot: D0AAZ5; Crystallized sequence: 63-232; N-ter (63-88) unobserved in the electron density (coverage 62%).

4kz1: VirB8 from *Bartonella grahamii*; Uniprot: C6AER9; Crystallized sequence: 51-222; N-ter (51-84) and C-ter (220-222) unobserved in the electron density; Coverage 61%.

4lso: VirB8 from *Bartonella quintana*; Uniprot: Q6FYW3; Crystallized sequence: 65-234; N-ter (65-93) unobserved in the electron density; Coverage 60%.

4mei: VirB8 from *Bartonella tribocurum*; Uniprot: A9IWN6; Crystallized sequence: 51-222; N-ter (51-84) and C-ter (221-222) unobserved in the electron density; Coverage 61%.

4nhf: VirB8 from *Bartonella grahamii*; Uniprot: C6AAT5; Crystallized sequence: 63-233; N-ter (63-88) and C-ter (233) unobserved in the electron density (coverage 61%).

4o3v: VirB8 from *Rickettsia typhii*; Uniprot: Q68X84; Crystallized sequence: 61-232; N-ter (61-89), loop β3-β4 (208-209) and C-ter (232) unobserved in the electron density (coverage 60%).

5aiw: TraH/Orf8 from *Enterococcus faecalis*; Uniprot: Q9AL12; NMR sequence: 56-183 (coverage 69%).

5cnl: IcmL-like from *Legionella pneumophila*; Uniprot: Q5ZZ91; Crystallized sequence: 22-176; C-ter (162-176) unobserved in the electron density (coverage 79%).

5i97: TraE from *Escherichia coli*; Uniprot: Q17U16; Crystallized sequence: 70-232; N-ter (70-87) and C-ter (231-232) unobserved in the electron density (coverage 59%).

6iqt: CagV from *Helicobacter pylori*; Uniprot: O25265; Crystallized sequence: 74-229; N-ter (74-85), loop β3-β4 (202-209) and C-ter (229) unobserved in the electron density (coverage 51%).

6zgn: OrfG from *Streptococcus thermophilus*; Uniprot: Q70CA4; Crystallized sequence: 65-204; N-ter (65-91), loop before β1 (134-138) and loop β2-β3 (169-175) unobserved in the electron density (coverage 31%).

Table S2a: Sequence comparison

		Gram-negative VirB8													Gram-positive VirB8-like			
		Class I^-				Class II^-						Class III^-			Class I^+			
		5i97	4akz	4nhf	4jf8	4mei	4kz1	4lso	2cc3	6iqt	4o3v	3wz4	3wz3	5cnl	6zgn	3ub1	4ec6	5aiw
5i97	length	143																
	ident.	1																
	simil.	1																
4akz	length	145	138															
	ident.	0.414	1															
	simil.	0.586	1															
4nhf	length	149	148	144														
	ident.	0.383	0.419	1														
	simil.	0.557	0.534	1														
4jf8	length	150	149	145	144													
	ident.	0.367	0.422	0.976	1													
	simil.	0.573	0.564	0.924	1													
4mei	length	150	145	149	150	136												
	ident.	0.200	0.338	0 282	0.287	1												
	simil.	0.413	0.51	0.456	0.473	1												
4kz1	length	149	144	149	150	136	135											
	ident.	0.215	0.340	0.295	0.300	0.956	1											
	simil.	0.436	0.535	0.456	0.473	0.971	1											
4lso	length	152	150	150	151	141	141	141										
	ident.	0.204	0.300	0.253	0.265	0.837	0.837	1										
	simil.	0.454	0.493	0.460	0.477	0.879	0.894	1										
2cc3	length	157	153	158	159	146	146	151	144									
	ident.	0.248	0.242	0.241	0.233	0.240	0.240	0.219	1									
	simil.	0.331	0.279	0.261	0.346	0.425	0.425	0.431	1									
6iqt	length	154	151	157	158	151	150	155	157	135								
	ident.	0.091	0.139	0.134	0.139	0.133	0.133	0.136	0.115	1								
	simil.	0.227	0.258	0.280	0.279	0.265	0.267	0.252	0.274	1								
4o3v	length	163	158	163	164	152	152	156	156	156	138							
	ident.	0.147	0.196	0.172	0.195	0.151	0.145	0.160	0.186	0.103	1							
	simil.	0.282	0.310	0.319	0.335	0.265	0.322	0.308	0.333	0.244	1							
3wz4	length	165	161	165	166	154	153	158	163	154	162	137						
	ident.	0.091	0.093	0.091	0.102	0.104	0.085	0.089	0.074	0.104	0.062	1						
	simil.	0.249	0.248	0.285	0.295	0.292	0.288	0.272	0.282	0.266	0.235	1						
3wz3	length	166	161	165	166	154	153	159	163	155	161	139	137					
	ident.	0.042	0.081	0.097	0.054	0.091	0.084	0.076	0.074	0.058	0.037	0.266	1					
	simil.	0.187	0.205	0.206	0.175	0.260	0.261	0.245	0.227	0.187	0.199	0.453	1					
5cnl	length	169	164	167	168	157	157	161	166	158	164	148	149	139				
	ident.	0.112	0.110	0.132	0.125	0.096	0.096	0.099	0.060	0.101	0.073	0.264	0.134	1				
	simil.	0.231	0.226	0.252	0.262	0.236	0.255	0.248	0.217	0.234	0.220	0.432	0.322	1				
6zgn	length	152	147	153	153	145	144	150	153	145	149	144	144	146	101			
	ident.	0.059	0.068	0.059	0.072	0.062	0.063	0.053	0.059	0.076	0.060	0.056	0.069	0.062	1			
	simil.	0.197	0.204	0.177	0.170	0.172	0.181	0.160	0.163	0.186	0.168	0.188	0.208	0.164	1			
3ub1	length	169	165	171	171	163	162	168	170	158	165	159	159	160	127	122		
	ident.	0.041	0.042	0.035	0.041	0.080	0.086	0.083	0.035	0.101	0.042	0.063	0.063	0.063	0.095	1		
	simil.	0.189	0.188	0.164	0.175	0.178	0.185	0.173	0.171	0.203	0.182	0.182	0.201	0.181	0.323	1		
4ec6	length	158	153	157	157	151	150	155	159	147	155	146	146	147	114	129	109	
	ident.	0.051	0.046	0.045	0.051	0.046	0.047	0.032	0.038	0.088	0.039	0.055	0.089	0.075	0.105	0.109	1	
	simil.	0.190	0.206	0.153	0.159	0.166	0.167	0.155	0.182	0.218	0.194	0.240	0.206	0.197	0.290	0.256	1	
5aiw	length	165	161	166	167	158	157	162	167	154	161	154	155	159	141	155	144	128
	ident.	0.067	0.087	0.108	0.096	0.063	0.064	0.056	0.066	0.065	0.118	0.065	0.071	0.031	0.028	0.077	0.049	1
	simil.	0.200	0.193	0.217	0.198	0.203	0.204	0.191	0.186	0.175	0.236	0.214	0.232	0.195	0.177	0.200	0.208	1

Table S2b : TM-scores

	Gram-negative VirB8													Gram-positive VirB8-like			
	Class I$^-$				Class II$^-$						Class III$^-$			Class I$^+$			
	5i97	4akz	4nhf	4jf8	4mei	4kz1	4lso	3cc3	6iqt	4o3v	3wz4	3wz3	5cnl	6zgn	3ub1	4ec6	5aiw
5i97	1																
4akz	0.9167	1															
4nhf	0.8942	0.9071	1														
4jf8	0.893	0.9259	0.9438	1													
4mei	0.8768	0.8754	0.8789	0.8699	1												
4kz1	0.8854	0.8842	0.879	0.8748	0.9948	1											
4lso	0.8665	0.8619	0.878	0.8702	0.9819	0.984	1										
2cc3	0.825	0.8901	0.8365	0.838	0.9123	0.9154	0.883	1									
6iqt	0.8129	0.8043	0.8053	0.7898	0.8311	0.8298	0.8377	0.8139	1								
4o3v	0.7696	0.7971	0.8158	0.7971	0.8208	0.8247	0.8242	0.8326	0.784	1							
3wz4	0.7218	0.7233	0.7432	0.7402	0.7669	0.7691	0.7674	0.7462	0.7733	0.739	1						
3wz3	0.7054	0.7159	0.7391	0.7371	0.7257	0.7291	0.7221	0.7282	0.7408	0.7414	0.8873	1					
5cnl	0.7108	0.7177	0.7397	0.7324	0.7519	0.75	0.7468	0.7258	0.7738	0.7559	0.8823	0.8142	1				
6zgn	0.733	0.7587	0.7454	0.7442	0.743	0.7445	0.7426	0.7586	0.7462	0.7548	0.7725	0.7626	0.7655	1			
3ub1	0.6146	0.6399	0.6202	0.6237	0.6259	0.6266	0.6342	0.6268	0.6432	0.656	0.6907	0.6641	0.6714	0.8122	1		
4ec6	0.6871	0.7121	0.7052	0.715	0.7119	0.7081	0.7212	0.6703	0.7231	0.6991	0.7363	0.716	0.7169	0.8057	0.7764	1	
5aiw	0.6882	0.6891	0.7054	0.6988	0.6775	0.6757	0.6976	0.6962	0.7257	0.7022	0.7033	0.7047	0.678	0.7036	0.6537	0.656	1

Table S2c: rmsd (Å)

	Gram-negative VirB8													Gram-positive VirB8-like			
	Class I$^-$				Class II$^-$						Class III$^-$			Class I$^+$			
	5i97	4akz	4nhf	4jf8	4mei	4kz1	4lso	3cc3	6iqt	4o3v	3wz4	3wz3	5cnl	6zgn	3ub1	4ec6	5aiw
5i97	0																
4akz	1.426	0															
4nhf	1.628	1.631	0														
4jf8	1.56	1.525	1.316	0													
4mei	2.059	1.837	1.738	1.812	0												
4kz1	2.062	1.818	1.754	1.758	0.326	0											
4lso	2.133	1.86	1.969	2.015	0.602	0.562	0										
2cc3	2.128	1.503	1.996	2.114	1.483	1.416	1.704	0									
6iqt	2.286	2.013	2.256	2.505	1.513	1.395	1.738	1.979	0								
4o3v	2.942	2.172	2.495	2.465	2.061	2	2.348	2.488	2.037	0							
3wz4	3.108	2.843	2.687	2.628	2.096	2.103	2.134	2.456	2.408	2.379	0						
3wz3	3.179	2.95	2.694	2.715	2.799	2.82	2.84	2.759	2.77	2.638	1.691	0					
5cnl	3.045	2.823	2.902	3.152	2.301	2.301	2.475	2.814	2.498	2.452	1.631	2.161	0				
6zgn	2.704	2.444	2.605	2.62	2.401	2.402	2.411	2.314	2.359	2.33	2.262	2.393	2.399	0			
3ub1	3.362	3.271	3.675	3.415	3.312	3.248	3.332	3.263	3.219	3.002	2.849	3.18	3.042	1.99	0		
4ec6	2.841	2.718	2.939	2.767	2.691	2.673	2.558	3.114	2.762	2.757	2.599	2.939	2.97	1.878	2.301	0	
5aiw	2.943	2.777	2.931	2.888	2.774	2.757	2.852	2.997	2.774	3.13	2.939	2.863	3.034	2.474	2.637	3.12	0

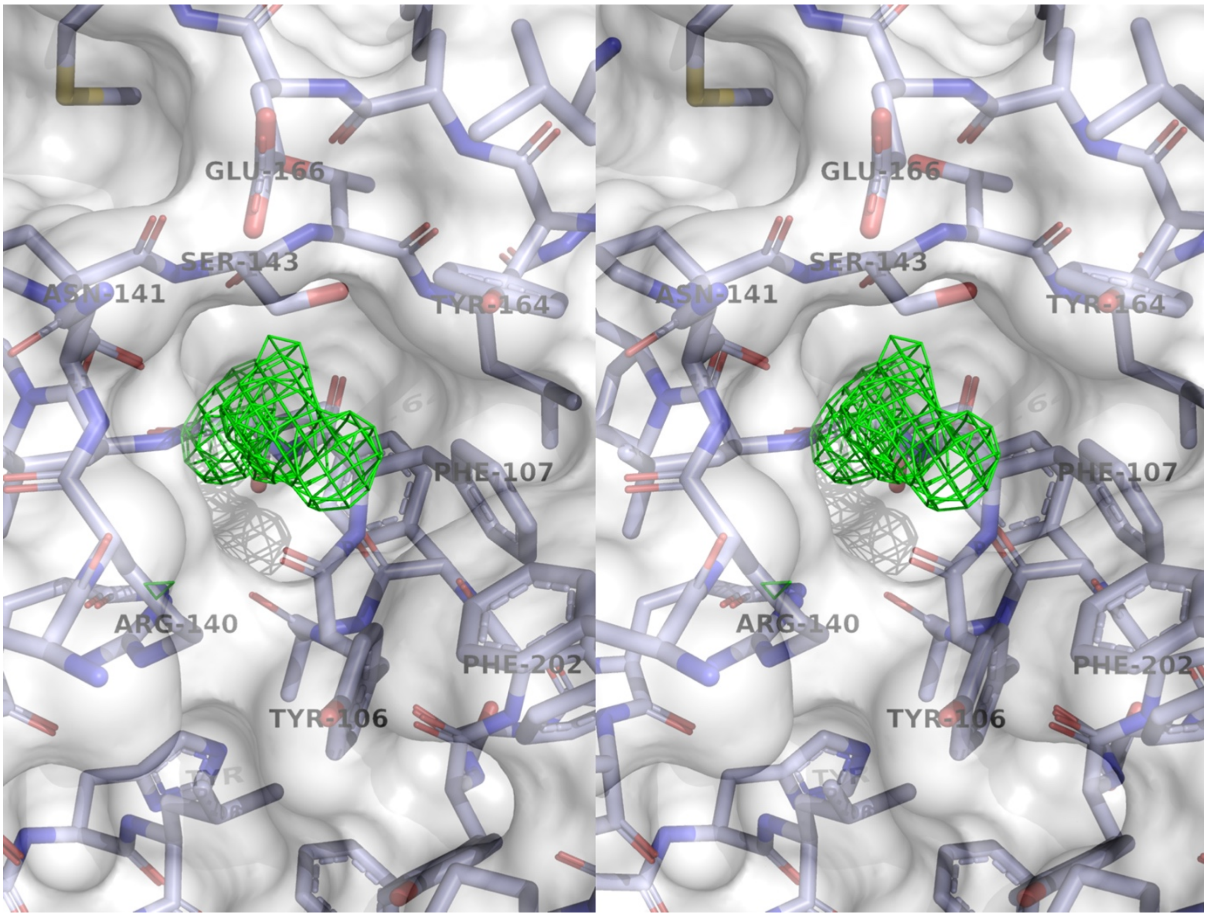

Figure S1: Presence of an unknown ligand bound to OrfG$_{64\text{-}204}$. Stereo view of an unattributed blob of electron density observed in the Fo-Fc map contoured at +3σ. Surrounding amino acids are shown in sticks and labeled.

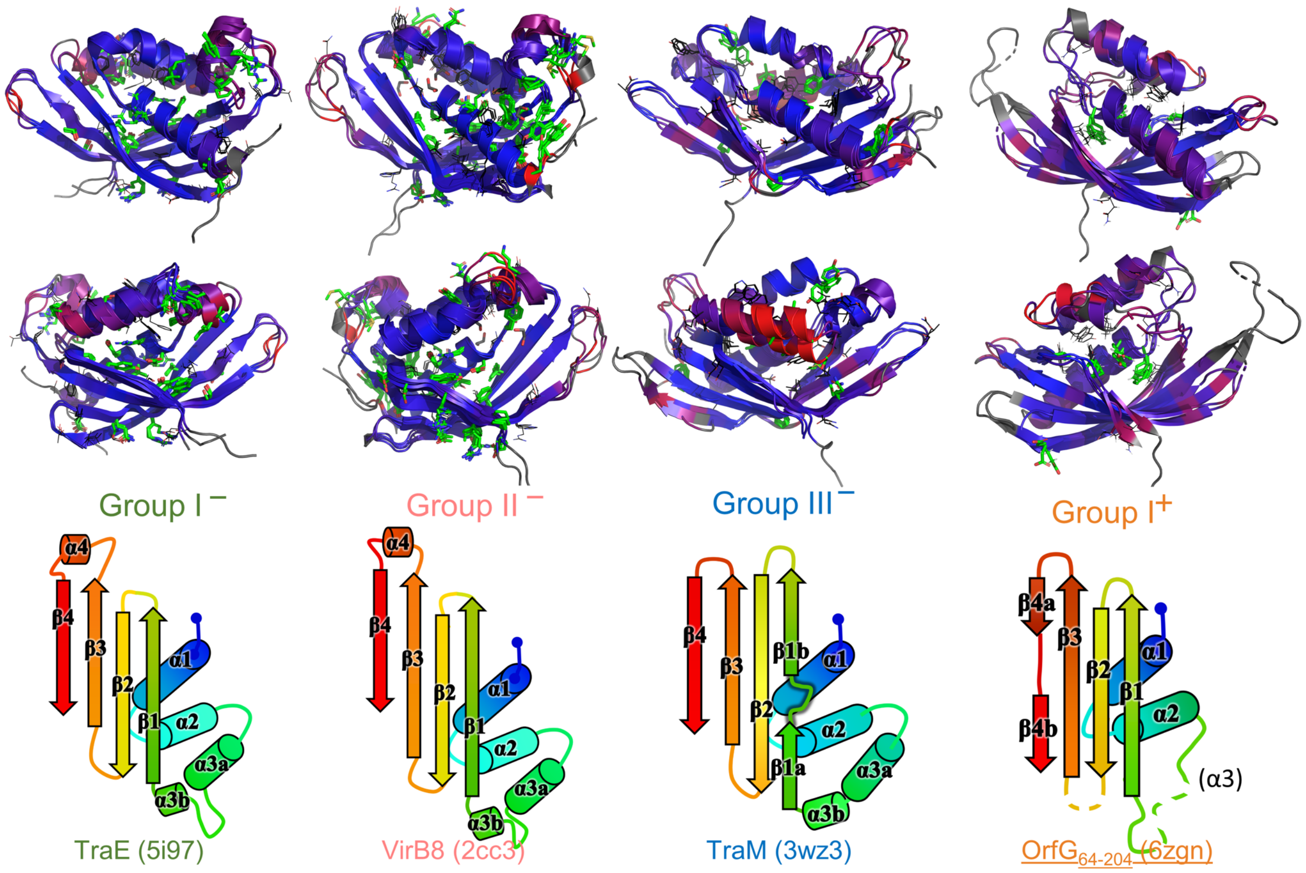

Figure S2: Superposition of VirB8-like structures within each class identified in the structural tree. Structures were superposed by mTM-Align, and represented using the same color code as in Figure 3 (see also Materials and Methods). Amino acid sidechains that are conserved are shown in green sticks, those that share a similar nature within a class are drawn as black lines. For each class, front view, rear view and secondary structure topology of a representative member are shown vertically.

References:

Dong R, Peng Z, Zhang Y & Yang J (2018) mTM-align: an algorithm for fast and accurate multiple protein structure alignment. *Bioinformatics* **34**: 1719-1725.

Dong R, Pan S, Peng Z, Zhang Y & Yang J (2018) mTM-align: a server for fast protein structure database search and multiple protein structure alignment. *Nucleic Acids Res* **46**: W380-W386.
